# Near-infrared fundus autofluorescence alterations correlate with swept-source optical coherence tomography angiography findings in patients with retinitis pigmentosa

**DOI:** 10.1038/s41598-021-82757-5

**Published:** 2021-02-04

**Authors:** Marco Nassisi, Carlo Lavia, Saddek Mohand-Said, Vasily Smirnov, Aline Antonio, Christel Condroyer, Serge Sancho, Juliette Varin, Alain Gaudric, Christina Zeitz, José-Alain Sahel, Isabelle Audo

**Affiliations:** 1 Sorbonne Université, INSERM, CNRS, Institut de la Vision, 17 rue Moreau, 75012 Paris, France; 2 CHNO des Quinze-Vingts, INSERM-DGOS CIC1423, 28 rue de Charenton, 75012 Paris, France; 3 Department of Clinical Sciences and Community Health, University of Milan, Milan, Italy; 4 Ophthalmological Unit, Fondazione IRCCS Ca’ Granda, Ospedale Maggiore Policlinico, Milan, Italy; 5 Université de Paris, Ophthalmology Department, AP-HP, Hôpital Lariboisière, 75010 Paris, France; 6 Surgical Department, Ophthalmology Service, Azienda Sanitaria Locale TO 5, 10023 Chieri, Italy; 7 Fondation Ophtalmologique Adolphe de Rothschild, 75019 Paris, France; 8 Department of Ophthalmology, University of Pittsburgh Medical School, Pittsburgh, PA 15213 USA; 9 Académie des Sciences-Institut de France, 75006 Paris, France; 10 Institute of Ophthalmology, University College of London, London, EC1V 9EL UK

**Keywords:** Hereditary eye disease, Retinal diseases

## Abstract

Thirty-eight patients from 37 families with retinitis pigmentosa (RP) underwent macular 6 × 6-mm swept-source optical coherence tomography angiography (SS-OCTA) and 30° near-infrared fundus autofluorescence (NIR-FAF) acquisitions in one eye. Superficial vascular complex (SVC), deep capillary complex (DCC) and choriocapillaris (CC) angiograms were registered with NIR-FAF acquisitions to comparatively assess subjects with and without central area of preserved NIR-FAF (APA). On the subset of patients showing an APA, the vessel densities for SVC and DCC and flow deficits for CC were assessed in three directions (superior, inferior and temporal) from the fovea and compared to healthy 1:1 age-matched controls. Nine patients with no APA had evidence of severe central OCTA alterations at all levels, especially in the DCC. In the other 29 subjects presenting APA, all OCTA parameters were similar to healthy eyes within the APA, where the retina preserves its structural integrity. Outside the APA, both the DCC and CC were significantly reduced in all directions. These alterations are probably related to the outer retinal atrophy outside the APA. Comparing OCTA to other imaging modalities is helpful to determine the potential interest of OCTA findings as an outcome measure for disease status and progression.

## Introduction

Retinitis pigmentosa (RP) comprises a group of genetically heterogeneous inherited retinal rod-cone dystrophies, characterized by progressive degeneration of the photoreceptors (PR) leading to night blindness, visual field constriction, and, eventually, central vision loss^1^. Mid-peripheral intraretinal bone spicule-like pigmentation, waxy/pale optic disc and generalized retinal vascular thinning represent the main fundoscopic findings associated with RP. Several approaches are being developed for treatment of RP, mostly depending on the genetic background of the disease^2^. These include gene augmentation/replacement therapy, gene editing, antisense oligonucleotides and cell therapy^2^. Clinical trials require solid and objective measurements to assess the disease stage and progression, and establish reliable outcomes for appropriate selection of candidates and evaluation of the success of the treatment.

In the recent years, optical coherence tomography angiography (OCTA) has provided new insights into retinal and choroidal microvasculature in many ocular diseases^3^. OCTA detects the blood flow using intrinsic signals to capture the location of blood vessels^3^. The high resolution of the swept source OCTA improved the assessment of the choriocapillaris (CC), allowing better correlations between the retinal vascular, structural and functional alterations in vivo. Several studies investigating the retinal microvascular abnormalities in RP patients using OCTA reported a decreased vascular density in all vascular plexuses, in particular in the para- and perifoveal regions^4,5^. However, very few of them tried to correlate these findings with data from other imaging modalities, while a multimodal approach would be crucial to contextualize the microvascular changes in RP.

**Figure 1:**
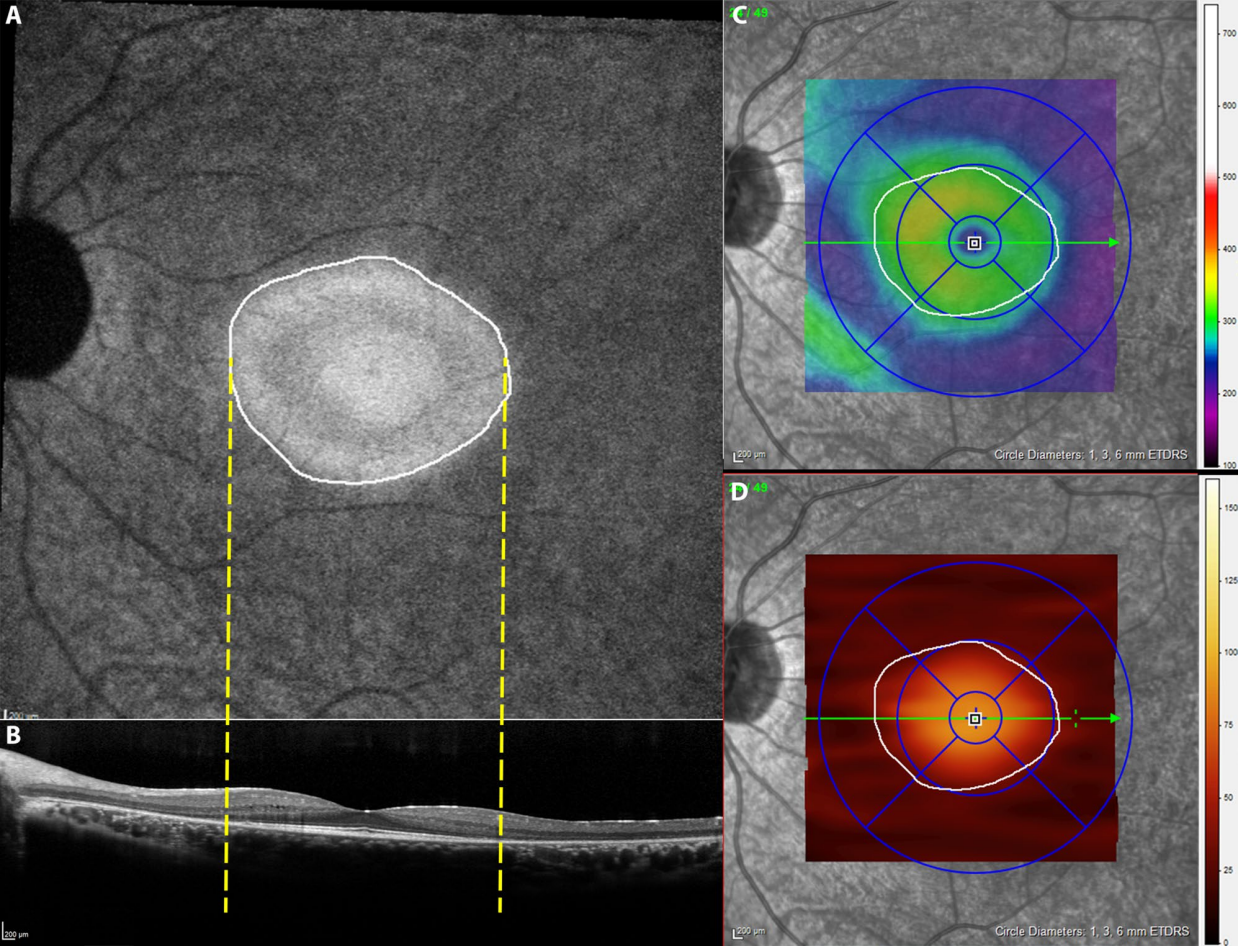
A 30° near-infrared fundus autofluorescence of a patient with retinitis pigmentosa (**A**). The limits of the area of preserved autofluorescence (white line) correspond to the area of preservation of the ellipsoid zone band on optical coherence tomography (OCT, yellow dotted lines, **B**). Roughly, outside this area (white line), the OCT thickness maps (**C**,**D**) show an important reduction of the overall retinal thickness (inner limiting membrane–Bruch’s membrane, **C**) and, more specifically, of the outer retinal thickness (external limiting membrane–Bruch’s membrane, **D**).

In order to shed further light on this matter, we correlated the swept source OCTA (SS-OCTA) images with near-infrared fundus autofluorescence (NIR-FAF), which provides an accurate information on the status of RPE in RP^6,7^ and often reveals a central area of preserved autofluorescence (APA). The border of the preserved APA (often hyperautofluorescent) roughly corresponds to the location where the ellipsoid zone (EZ) on optical coherence tomography (OCT) disappears and the outer retinal layers are significantly thinner (Fig. 1)^8^. Inside this area, the cone-mediated visual function is relatively preserved^9^. In previous studies, NIR-FAF demonstrated to have better correlations with OCT findings than SW-FAF^8,10^. Furthermore, measurements on NIR-FAF showed higher reliability because of the higher contrast between the APA and the damaged tissue on the outside, giving a good approximation of the remaining healthy retina^8^. Hence, NIR-FAF represents a relevant tool in assessing the disease progression towards the center of the macula and in correlating the RPE/PR loss with the retinal vascular changes^10^.

## Methods

Patients with a clinical diagnosis of RP were recruited at the Centre Hospitalier National d'Ophtalmologie des Quinze-Vingts, Paris, France, between January and September 2019. Patients with high myopia (> 6 Diopters), high hyperopia (> 3 Diopters), or any other ocular disease were excluded from the study. Eyes with visually non-significant vitreoretinal interface disease, such as a subtle epiretinal membrane only detectable on SD-OCT, or with subtle small intraretinal cysts (not involving the fovea), were not excluded. When both eyes were eligible, the right eye was chosen for analysis. Informed consent was obtained from all included subjects (patients and relatives). For minors (age < 18), informed consent was obtained from both parents and/or legal guardians. The study protocol was in accordance with the principles of the Declaration of Helsinki and was approved by a national ethics committee (CPP, *Comité de Protection Des Personnes*). When possible, blood samples from the patients and their relatives were collected for genetic screening, which was performed at the Institut de la Vision, Paris, France, as previously reported^11–13^.

### Patients

Thirty-eight patients (37 families, 17 females, mean age 40.9 ± 16.3 years, range 12–82 years) with RP (including sectorial form) were enrolled. The best corrected visual acuity was 0.21 ± 0.27 logarithm of the minimum angle of resolution (LogMar; median 0.10 LogMar, 20/25 Snellen equivalent). Of all enrolled patients, 22 (57.89%) had molecular diagnosis: 9 were related to mutations in *USH2A*, 2 to *RPGR*, 1 to *INPP5E*, 1 to *RPE65*, 2 to *MYO7A*, 2 to *RHO* (sectorial forms), 1 to *PRPH2*, 1 to *AIPL1,* 1 to *NR2E3*, 1 to *EYS* and 1 to *MAK * (supplemental material).

### Images acquisition

All patients underwent SS-OCTA examination with 100 kHz Plex Elite 9000 (Carl Zeiss Meditec Inc., USA) which operates at 100,000 A-scan per second with an axial resolution of approximately 5 µm in tissue. Compared to spectral domain devices, the higher light wavelength of this instrument [i.e. 1050 nm centrally (1000–1100 nm full bandwidth)] ensures a higher tissue penetration. After pupil dilatation, each patient underwent a 6 × 6-mm (500 A-scans × 500 B-scans) OCTA scan acquisition on the fovea. Only scans with sufficient image quality (signal strength index > 7/10) were retained for the study^14,15^. Manufacturer’s fully-automated retinal layer segmentation algorithm was applied to the three-dimensional structural OCT data in order to segment the superficial vascular complex (SVC) slab (between the inner limiting membrane (ILM) and inner plexiform layer–inner nuclear layer (IPL–INL) interface), the deep capillary complex (DCC) slab (between the IPL–INL and outer plexiform layer–outer nuclear layer (OPL–ONL) interfaces) and the CC slab (20 µm thick starting from the Bruch’s membrane)^16,17^. In case of segmentation errors, the segmentation was manually refined and verified by a retina expert (IA). This segmentation was applied to OCTA flow intensity data to obtain vascular images. Maximum projection analyses of the flow intensity were performed to generate “en-face” images of the SVC, DCC and CC plexuses. Projection artifacts were automatically removed using the software of the machine on the DCC and the CC angiograms. The same eye then underwent a NIR-FAF acquisition (30° of field, 787 nm of excitation; HRA II, Heidelberg Engineering, Germany). The image had to be centered on the fovea and was the result of at least 30 averaged single frames obtained through an automatic real time (ART) module.

### Image processing

**Figure 2:**
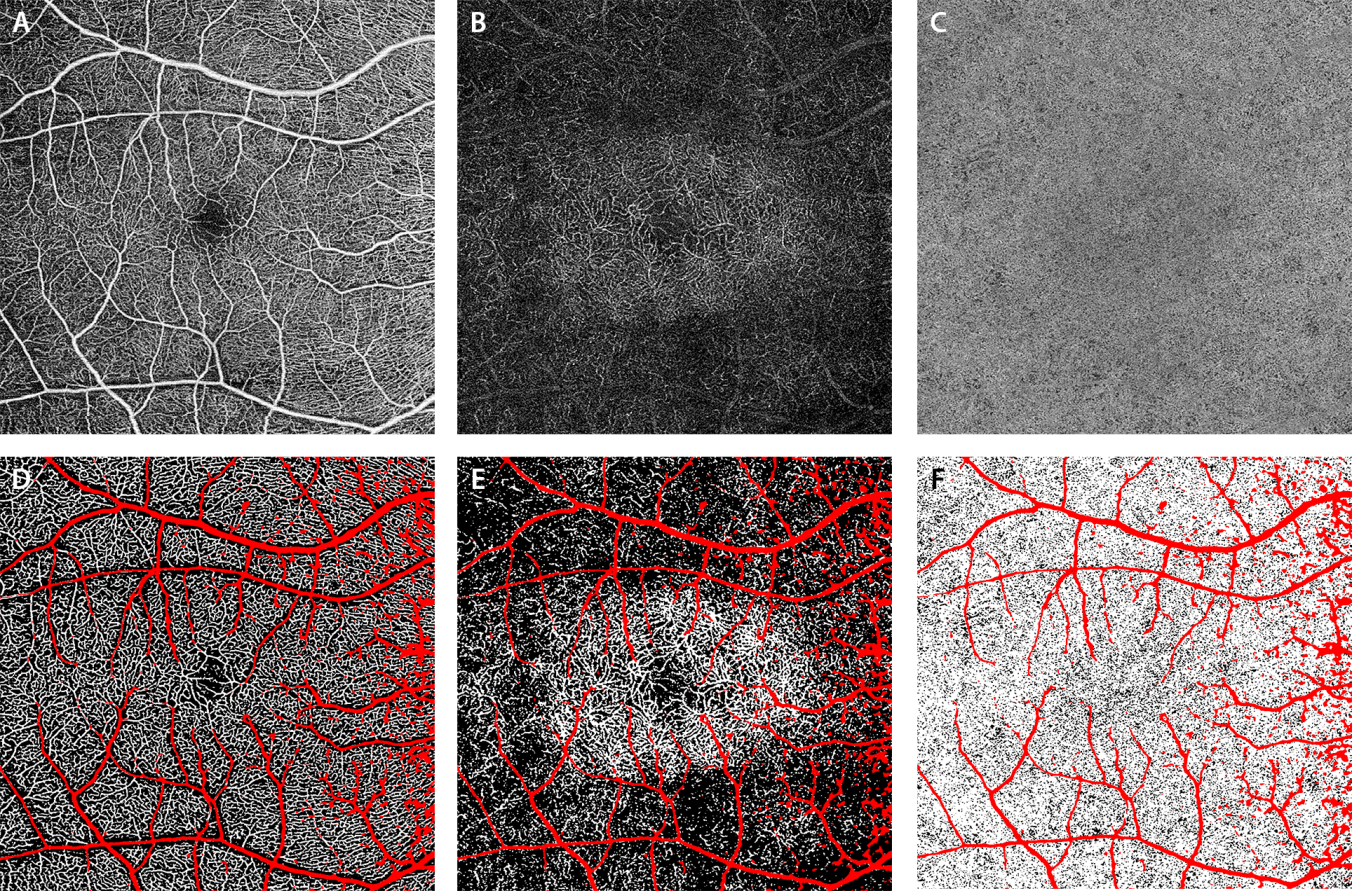
Optical coherence tomography angiography (OCTA) of a patient with retinitis pigmentosa, showing the superficial vascular complex (SVC, **A**), the deep capillary complex (DCC, **B**) and the choriocapillaris (CC, **C**). All angiograms were binarized in order to quantify the vessel density (white pixels) in the SVC (**D**) and DCC (**E**) and the flow deficits (black pixels) in the CC (**F**). Larger vessels were excluded from the analysis of all plexuses (red pixels).

**Figure 3:**
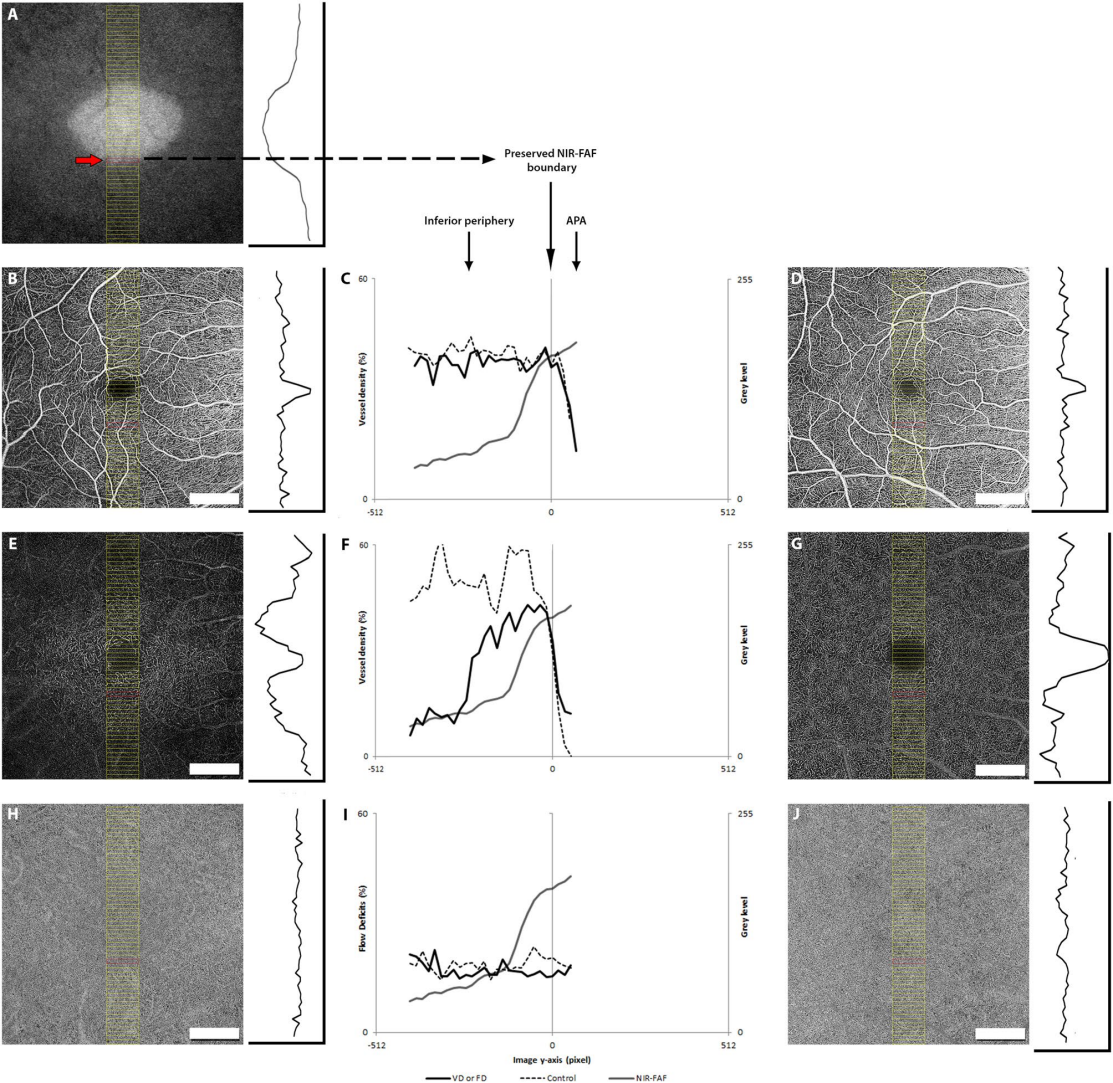
Method for quantitative analysis of near-infrared autofluorescence (NIR-FAF) and optical coherence tomography angiography used in the study. The analysis was performed in a series of consecutive 0.1 × 0.8-mm sample areas passing through the fovea (yellow rectangles in **A**, **B**, **D**, **E**, **G**, **H** and **J**) and spanning in three directions: superior, inferior and temporal (the latter not shown). The NIR-FAF image of the affected subject (**A**) was used to evaluate the intensity level (IL) profile, while the angiograms were used to quantify the vessel density (VD) of the superficial vascular plexus (SVC, **B**), the deep capillary complex (DCC, **D**) and the flow deficits (FD) of the choriocapillaris (CC, **F**). For each direction (superior, inferior and temporal), a graph was built where the IL (from **A**) was superimposed with the VD (**C**,**F**) or the FD (**I**) of the affected subject and of an age-matched control (black dotted lines in **D**, **G** and **J**). The preserved NIR-FAF (APA) boundary (which corresponded to the sharp decline of the IL in NIR-FAF, red arrow in **A** and red rectangles in **A**, **B**, **D**, **E**, **G**, **H** and **J**) was taken as “0” in the x-axis in order to allow the averaging of the data from the entire cohort.

All images were imported into ImageJ V.1.50 (National Institutes of Health, Bethesda, Maryland, USA; available at http://rsb.info.nih.gov/ij/index.html) to be analyzed^18^. The three OCT angiograms were registered with NIR-FAF acquisitions using the superficial vessels as references. A qualitative evaluation was first performed by two retina specialists (MN and CL) comparing subjects with and without central area of preserved NIR-FAF. In the subset of patients in which the APA was smaller than the 6 × 6-mm frame of the OCTA, a quantitative analysis was also performed. The OCT angiograms of the three vascular plexuses were binarized using previously reported methods^15,16,19^. Briefly, the SVC and DCC images were first processed with top-hat filter (window size, 12 pixels) and then duplicated to obtain two distinct binarized images. One was processed with a Hessian filter, followed by a global thresholding using the Huang’s fuzzy thresholding method, and the other (duplicate) image was binarized through a median local thresholding. Last, the two binarized images were combined to generate the final image in which were included only the pixels that existed on both binarized images. For the CC angiogram, the image was binarized using a global threshold. This threshold was determined by one standard deviation (SD) from a normal database (20 subjects at 20–39 years old) as previously described^19^. Areas presenting larger vessels were excluded from the vessel density (VD) and flow deficits (FD) analyses by applying a mask that used an intensity-based thresholding algorithm applied to the SVC C-scan (Gaussian window of 15 × 15 pixels, intensity threshold of 0.4, Fig. 2). Then, flow pixels in the SVC and DCC and non-flow pixels in the CC were counted for each C-scan in a series of 0.1 × 0.8-mm sample areas and divided by the total number of pixels to obtain the VD and the FD, respectively. Sample areas that were covered by the larger vessel masks (> 40% of the area) were excluded from the analysis. The series of sample areas spanned in three directions (superior, inferior and temporal) from the fovea. The nasal sector was not assessed since the presence of the optic nerve limited the extension of the analysis, precluding its accuracy. The resulting profiles were matched with the intensity level of the corresponding NIR-FAF acquisition, which was analyzed in the same regions. Given the heterogeneous surface of the APA across the cohort, data from all subjects were averaged using the APA edges as reference (i.e. the value from the sample area containing the border was set as “0” to build the profiles, Fig. 3). Finally, these profiles were compared with the ones resulting from healthy 1:1 age-matched controls (Fig. 3): a Wilcoxon signed rank test was performed to compare sample areas which were equidistant from the edges of the APA. The p-values were then plotted with the distance from the edge of the APA and the difference between the profiles was considered significant at least five consecutive p-values were < 0.05. All figures in this manuscript were created using Adobe Photoshop CC 2017 (Adobe, Inc., San Jose, CA, USA).

## Results

### Qualitative analysis

**Figure 4:**
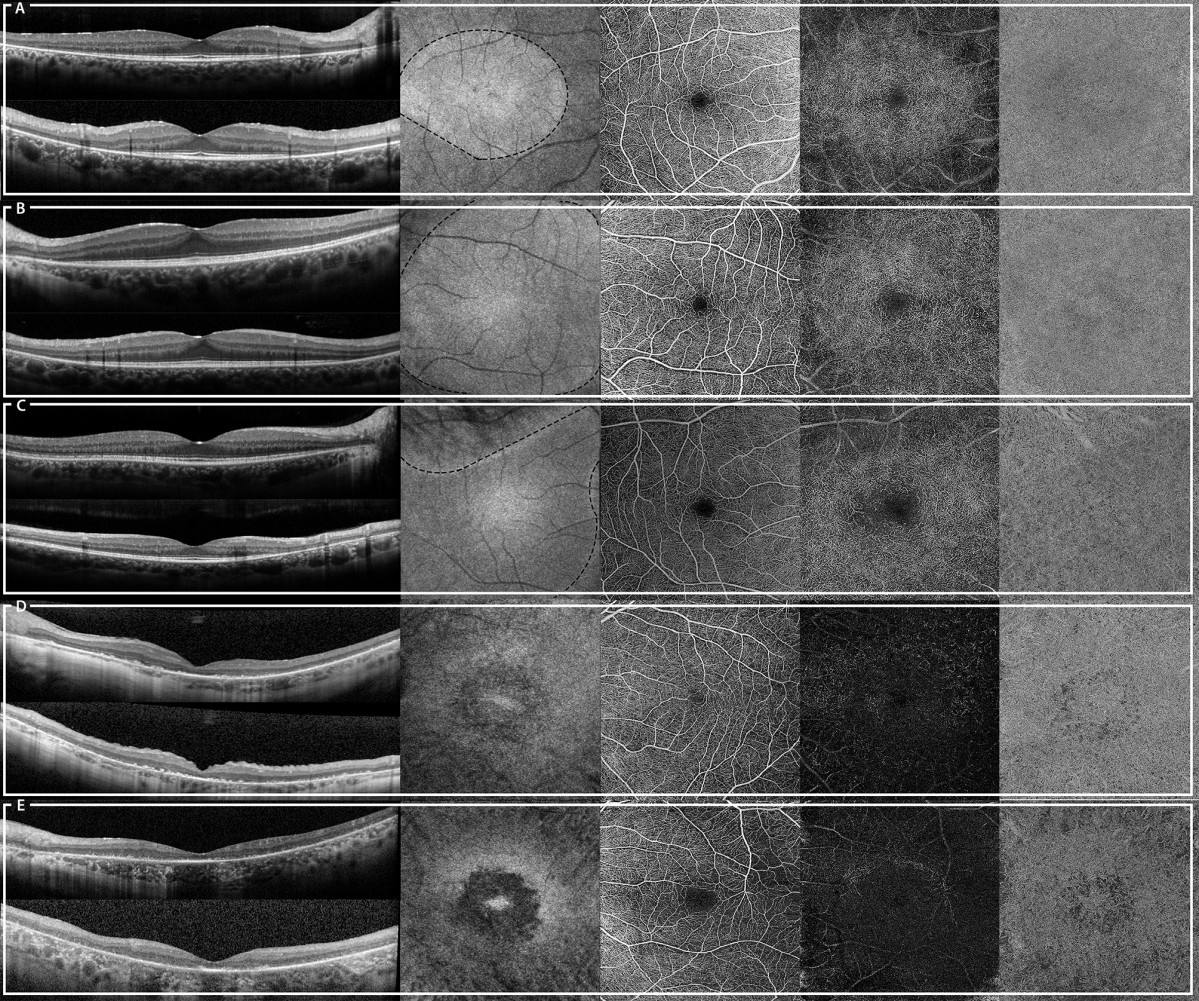
Images from patients with Retinitis Pigmentosa enrolled in the study. From the left to the right, a foveal optical coherence tomography (OCT) horizontal (top) and vertical (bottom) B-scans, a near-infrared fundus autofluorescence (NIR-FAF), the superficial vascular complex (SVC), the deep capillary complex (DCC) and the choriocapillaris (CC) angiograms are displayed. In the NIR-FAF image of (**A**–**C**), the black dotted lines mark the edge of the area of preserved NIR-FAF. This area is not recognizable in (**D**) and (**E**). Genetic data of displayed patients: CIC10938 (**A**): unknown; CIC12075 (**B**): unknown; CIC04704 (**C**): *RHO*; CIC09779 (**D**): *RPGR-ORF15;* CIC01532 (**E**)*: USH2A.*

**Figure 5:**
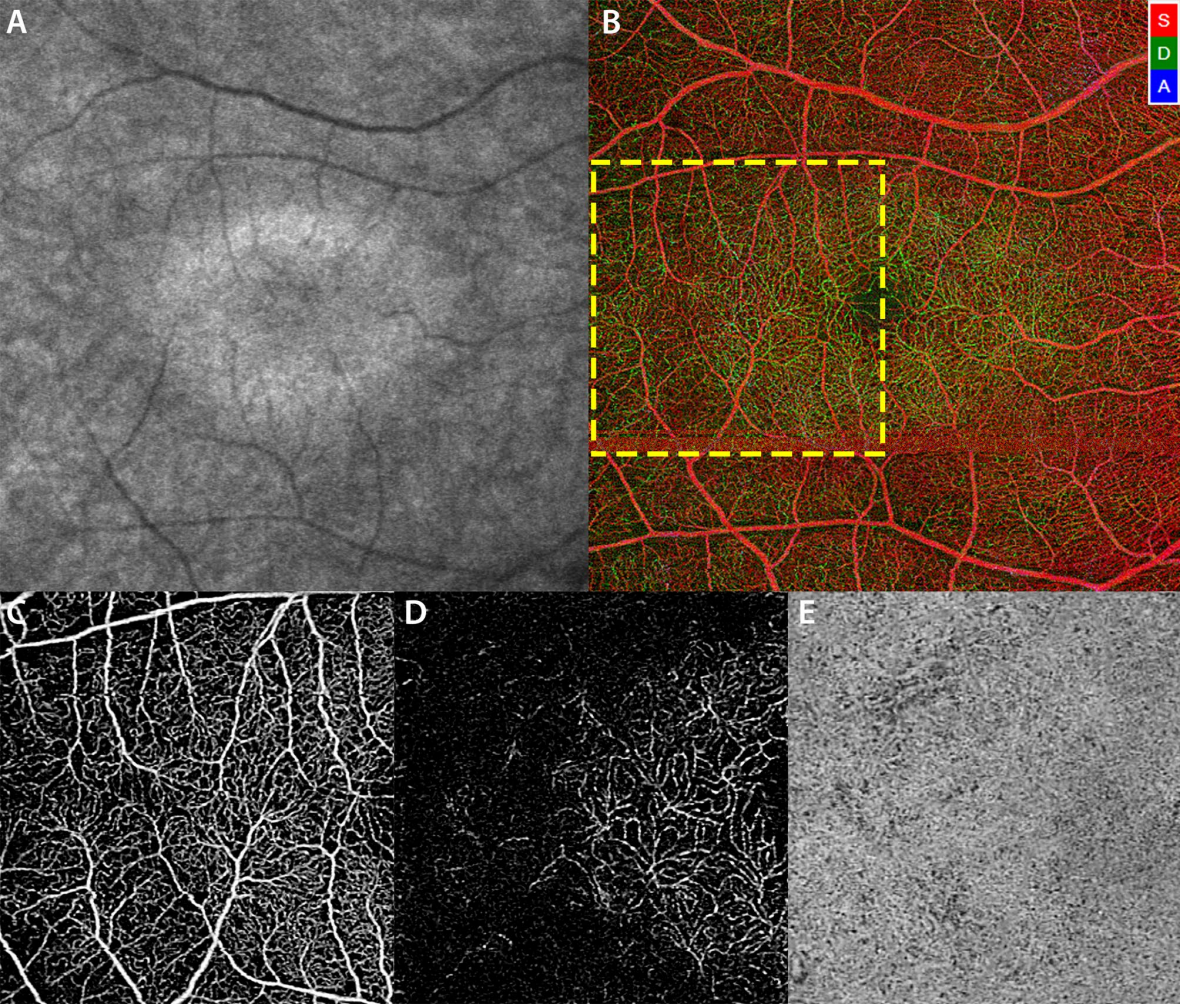
Patient affected by *USH2A*-related Retinitis Pigmentosa. The near-infrared fundus autofluorescence (NIR-FAF, **A**) shows a central area of preserved autofluorescence (APA) which, on optical coherence tomography angiography (OCTA), corresponds to an area where the retinal vasculature looks intact (**B**, color-coded map: red for superficial capillaries and green for deep capillaries). A 3 × 3 mm OCTA acquisition was taken on the edge of the APA (yellow box in **B**), to better show the vascular alterations in the immediate surroundings of the APA, at the level of the superficial vascular complex (SVC, **C**), the deep capillary plexus (DCC, **D**) and the choriocapillaris (CC, **E**). Outside the APA there is a mild capillary drop out in the SVC (**C**), while the DCC is completely depleted (**D**). Flow deficits in the CC are larger and more numerous outside the APA (**E**).

**Figure 6:**
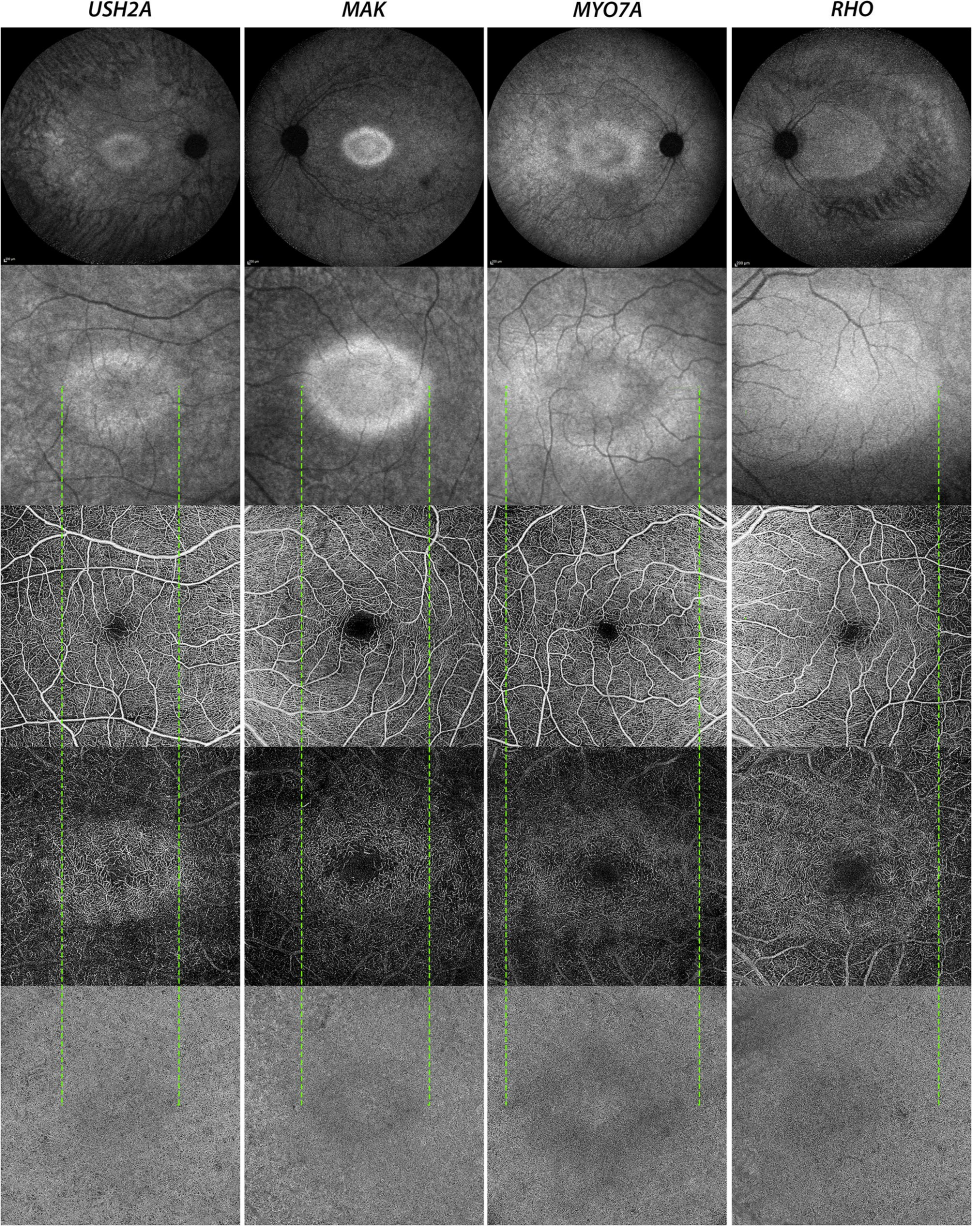
Near-infrared fundus autofluorescence (NIR-FAF, 55° of field top-first image and central 6 × 6 mm top-second image) and optical coherence tomography (OCT) angiograms [superficial and deep capillary plexuses (top-middle and bottom-middle) and choriocapillaris (bottom)] of patients with retinitis pigmentosa from different genetic backgrounds (*USH2A, MAK, MYO7A* and *RHO*). The vascular alterations outside the NIR-FAF preserved areas, at the level of the DCC and the CC are unrelated to the different genotypes. However, for the CC there was a slightly different degree of involvement. The yellow dotted lines indicate the margins of the area of preserved NIR-FAF. The variable NIR-FAF aspect of the APA with foveal hyperautofluorescence surrounded by hypoautofluorescence in USH2A-, MAK- and MYO7A-related patients or with an isoautofluorescence in the RHO-related patient is physiologic and it is related to the different concentrations of melanosomes in each subject.

In 9 patients, no autofluorescence at NIR-FAF was present in the posterior pole, corresponding to an outer retinal atrophy on OCT B-scans. These patients presented some degree of capillary drop out in the SVC, an almost depleted DCC and a diffuse increase of FD in the CC (Fig. 4E). The other 29 patients presented an APA within the limits of the 6 × 6-mm angiogram. In these cases, the DCC looked intact within the APA, while outside its density was markedly reduced and the typical capillary vortices were not recognizable (Figs. 4A–D, 5). This was especially obvious in sectorial RP where the edge of the vascular abnormalities in DCC followed the irregular borders of the APA (Fig. 4C). Qualitative assessment of the CC revealed similar changes. In general, the CC looked preserved within the APA, while it showed more and larger FD outside. These changes were particularly marked where the outer retinal atrophy was associated with retinal pigment epithelium (RPE) atrophy (which was detected on OCT B-scans as regions of hypertransmission, with disruption of the RPE and overlying outer retinal alterations). Interestingly, we did not notice any visible differences across various genetic backgrounds. (Fig. 6).

### Quantitative analysis

**Figure 7:**
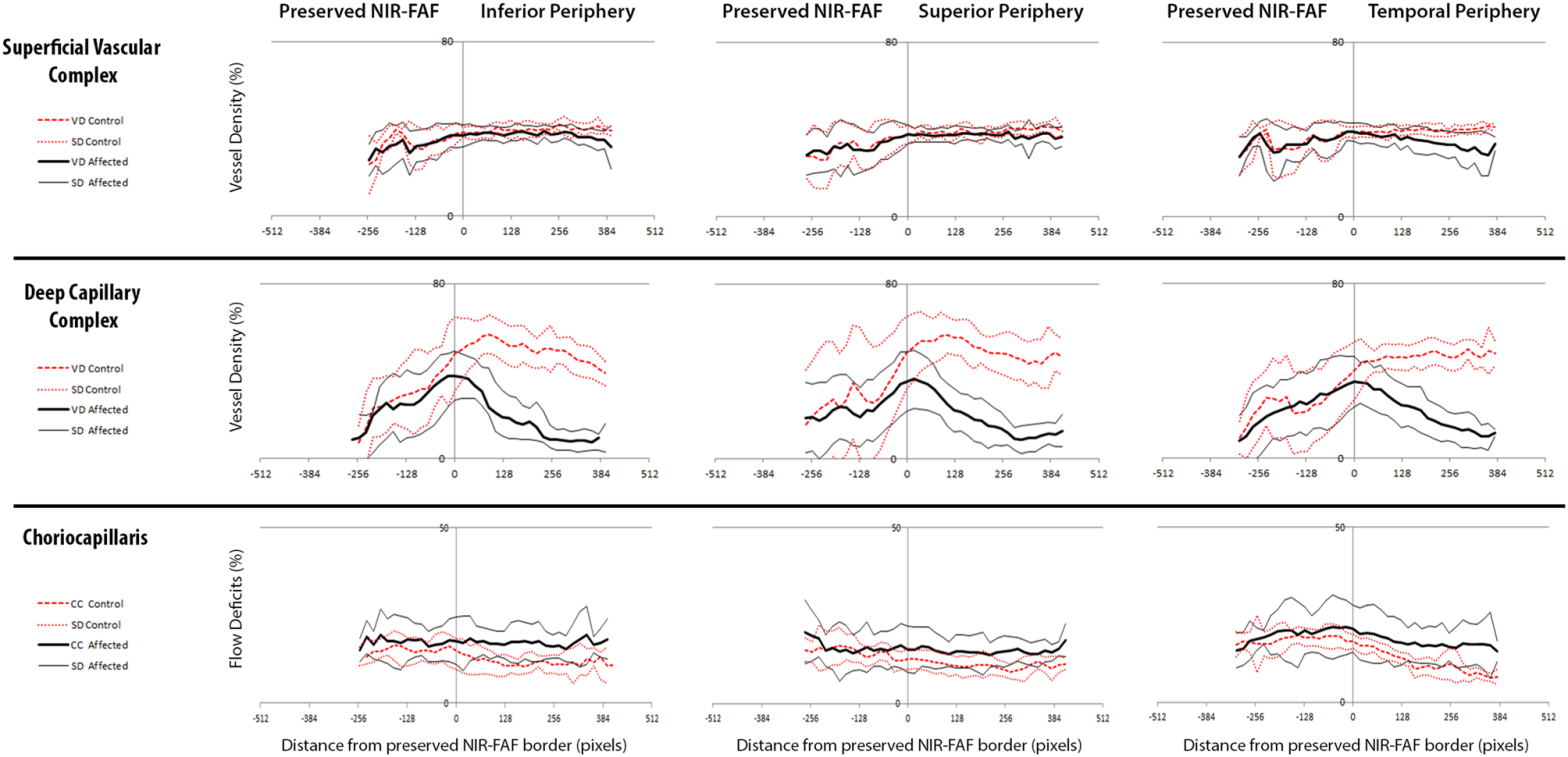
Vessel density (VD) and flow deficits (FD) profiles in patients with retinitis pigmentosa and age-matched controls, analyzed in three vascular layers, in three directions: superior, inferior and temporal from the fovea. The “0” on the x-axis corresponds to the border of the preserved near infrared fundus autofluorescence (NIR-FAF).

**Figure 8:**
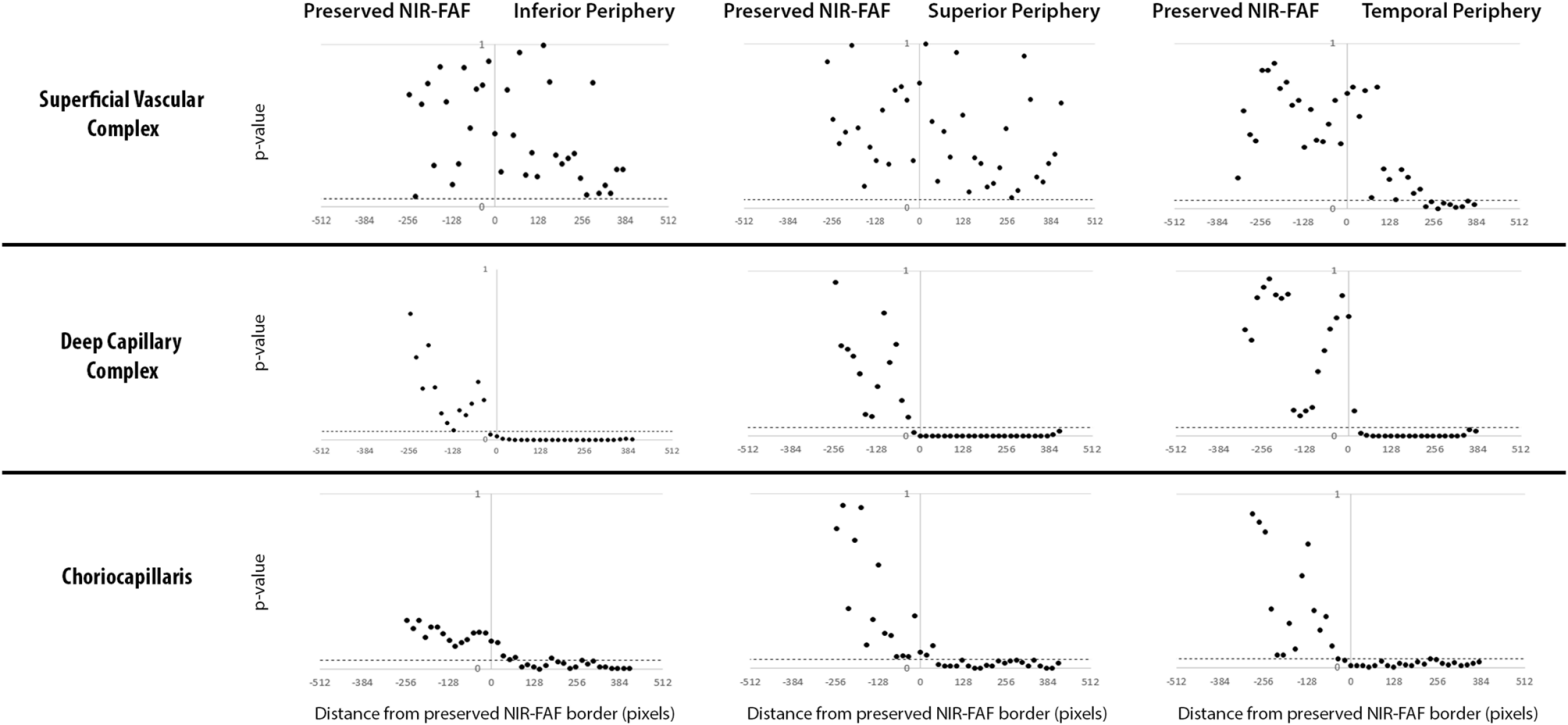
P-values obtained for each analyzed sample area from the comparison between patients with retinitis pigmentosa and age-matched controls. A Wilcoxon signed-rank test was performed to obtain each p-value. The black dotted line corresponds to the threshold of significance considered in the study (p < 0.05). The “0” on the x-axis corresponds to the border of the preserved near infrared fundus autofluorescence (NIR-FAF).

A quantitative assessment of the microvasculature was performed in the 29 subjects for whom the borders of APA fell inside the central 6 × 6-mm OCTA. No differences were found within the NIR-FAF APA, at any capillary level between RP patients and healthy controls (Figs. 7, 8). However, the mean VD in the SVC was decreased temporally outside the APA, by 2.91 ± 3.91% (range − 2.99 and 11.91%) compared to controls (calculated as mean VD in controls minus mean VD in affected for each sample area beyond the border of the APA). This difference becomes statistically significant after around 256 pixels (~ 1.5 mm) beyond the edge of the APA (Fig. 8). The VD in the DCC was significantly reduced outside the APA in all directions: the mean difference of VD compared to controls was 30.55 ± 9.1% (range 10.12 and 40.9%) inferiorly, 30.72 ± 7.25% (range 13.15 and 37.68%) superiorly and 25.35 ± 10.39% (range 4.89 and 39.25%). Finally, the FD in the CC (calculated in the same way as for VD) were slightly and significantly larger in RP patients in all directions outside the APA: the mean difference of FD was − 5.61 ± 1.18% (range − 7.74 and − 3.5%) inferiorly, − 3.98 ± 1.19% (range − 6.87 and − 2%) superiorly and − 5.82 ± 1.99% (range − 9.26 and − 3.5%) temporally (Figs. 7, 8).

## Discussion

In this study, using a SS-OCTA, we were able to document macular microvascular changes in patients with RP and correlate them with NIR-FAF findings. According to previous studies, the APA in NIR-FAF represents a region where the outer retinal layers and the EZ band on OCT are preserved, resulting in an island of preserved PR sensitivity and vision^8,10^. The absence of the APA on NIR-FAF usually denotes an advanced stage of disease in which we have found that all retinal capillary layers and CC are impaired on OCTA, even in the foveal center (Fig. 4E). We also demonstrated that eyes with earlier stages or milder forms of RP, with a persistent APA on NIR-FAF, preserve in this area normal retinal capillary layers and CC, both qualitatively and quantitatively. In contrast, outside the APA, retinal capillary density is decreased, especially at the DCC level. In healthy eyes, the DCC density moderately decreases towards the periphery, following the decrease of the ONL thickness and PR density^20,21^. The DCC is supposed to supply oxygen to the outer half of the INL and OPL^22^. However, several in vivo studies suggest a role of the DCC also in the PR oxygenation, even though this is still debated^22,23^. It is possible that the progressive loss of PR in RP could cause a decrease in the cellular oxygen demand, leading to a secondary vascular remodeling and attenuation of the DCC.

Data regarding the SVC density in patients with RP are currently under discussion. While several studies report an impairment of the SVC, mostly in the perifoveal sectors, no alterations were found in a recent study by Hagag et al.^5^. Unlike Hagag et al., we observed a mildly reduced VD of the SVC in the temporal sectors. The reason could be that we measured VD beyond the NIR-FAF “demarcation line” between preserved and altered outer retina and not globally in the temporal sector which could integrate areas of both preserved and absent PR. It is possible that outside the APA, with the loss of PR, enough O_2_ is provided to the inner retina and this results in a decrease of the SVC density as already suggested^24^, despite the concomitant decrease of the DCC density.

The reason why this decrease in VD was only found in the temporal part of the posterior pole remains unclear. One explanation could be the topographic distribution of the radial peripapillary capillary plexus (RPPC), which is not noticeable in the temporal sector of the macula compared to the inferior and superior sectors. The RPPC contributes to the SVC together with the superficial capillary plexus (SCP) and is usually preserved in RP as is the thickness of the retinal nerve fiber layers^25,26^. In the 6 × 6-mm OCTA, the SVC density measures globally the density of both the SCP and RPPC, hence, the decrease of the SVC density in the superior and inferior sectors could be masked by the normal density of the RPPC. Further investigations are needed to confirm this hypothesis.

The CC outside the APA presents only mild changes which vary among the eyes in contrast with the decrease observed in the SVC and DCC density that is present in all eyes. In particular, FD appear more numerous and larger in the areas where both the RPE and the outer retinal layers are atrophic (Fig. 4D,E). This seems coherent with the fact that the RPE secretes a variety of growth factors that are crucial for the preservation of the underlying CC^27^. Whether the CC impairment is present or not in RP patients is another debated issue, with evidence pointing both ways^28–33^. One possible explanation to this discrepancy was proposed by Hagag et al. who suggested that the CC alterations might be related to the underlying genetic defect and could specifically depend on whether it implies a direct damage to the RPE or only indirect alteration through primary PR degenerations^4^. While we agree that the genotype might contribute to the CC alterations, we did find evidence of CC impairment also in *USH2A*-related RP patients (Fig. 6), while the usherin protein is specifically localized in the PR with a presumed secondary RPE alteration. These inconsistent results could be attributed to the use of different OCTA machines, the SS-OCTA being better suited for analyzing the CC with its wavelength allowing a deeper tissue penetration. On the other hand, we recognize that the number of genetically characterized RP patients in our study was limited to allow comparisons between the different genotypes. However, we believe that in the context of a progressively degenerative disease, the RPE inevitably becomes dystrophic and atrophic whether directly or indirectly, and this might lead to CC attenuation. Further investigations including longitudinal data and a larger cohort of patients are required to confirm this hypothesis. Contrarily to our findings, previous studies reported an important reduction of choroidal blood flow in RP using different modalities such as Laser Doppler flowmetry (26% reduction of the choroidal blood flow), Laser speckle flowgraphy and magnetic resonance imaging (reduction of 75% and 52% respectively, in retinal and choroidal blood flow together), even in patients with preserved visual acuity^34–36^. However, unlike SS-OCTA, these techniques are not able to selectively give information about the CC. On the other hand, OCTA does not provide any parameter about the flow velocity; in fact even if the flow is reduced but still in the range of detectability, it is present in the angiogram.

This study has some limitations including the relatively small number of enrolled patients, the cross-sectional setting and the use of NIR-FAF, instead of short-wavelength fundus autofluorescence (SW-FAF), for the correlation with OCT and OCTA scans. SW-FAF is currently considered the gold standard for monitoring RP patients; however, this is mostly due to the more frequent availability of SW-FAF in clinical settings. The NIR-FAF findings are highly correlated with SW-FAF findings. In both modalities the border of the APA is often hyperautofluorescent and the location of its outer border has a good correspondence between SW-FAF and NIR-FAF images^8^. This hyperautofluorescent ring may be the result of a window defect secondary to a local rarefaction of the EZ band^37^. However, another hypothesis was advanced: in SW-FAF the ring may be indicative of an accelerated synthesis of lipofuscin to which the dysfunctional photoreceptors do not correspond with a sufficiently efficient detoxification^8,37^. This overload of lipofuscin may displace the physiologic localization of the melanosomes in the RPE which eventually cause the higher signal in NIR-FAF^8^. Intriguingly, Duncker et al. demonstrated that the inner border of this hyperautofluorescent ring is closer to the fovea in NIR-FAF and corresponds to an area where the EZ band on OCT appears to be intact^8^. At the same time, the same group demonstrated that the thickness values of the outer retinal layers (measured from Bruch’s membrane to the inner limit of the outer plexiform layer) were significantly decreased at distances that were closer to the fovea than the inner border of the hyperautofluorescent ring in SW-FAF^10^. Therefore, it is likely that not only NIR-FAF alterations precede the SW-FAF alterations but they are also better correlated with OCT findings. Furthermore, NIR-FAF provides an important advantage over SW-FAF imaging in RP: there is an abrupt loss of the NIR-FAF signal between the healthier and damaged retina outside the APA^8^. This sharp demarcation was very useful for the quantitative analysis that we performed for correlating the OCTA findings within and outside the APA. Nevertheless, our study has major strengths, including the use of a SS-OCTA machine, which provides high resolution angiograms and greater tissue penetration for CC visualization, and the recruitment of 1:1 age-matched controls to eliminate the confounding factor of age in all OCTA meaurements^38,39^.

In conclusion, our study emphasizes the importance of the contextualization of retinal microvascular changes in RP through multimodal imaging modalities. In previous publications, the vascular abnormalities in RP were arbitrarily studied using non-customized areas (e.g. para- or perifoveal sectors). The use of the latter might give misleading results when the amount of residual healthy retina is not taken into account.

Furthermore, as the APA represents an area of preserved outer retina and as it correlates well with OCTA, our findings suggest that the decrease of the retinal capillaries (above all in the DCC) and increase in CC FD constitute an adaptation to the outer retinal atrophy and loss of PR resulting from the reduction of the metabolic demand. Longitudinal studies are required to better understand the sequence of events and to confirm the value of OCTA parameters as relevant biomarkers in these patients.

## Supplementary Information

Supplementary Information.

## Data Availability

All relevant data are present in the manuscript.
