# Supplementary material for: Near-infrared fundus autofluorescence alterations correlate with swept-source optical coherence tomography angiography findings in patients with retinitis pigmentosa
Source: Sci Rep. 2021 Feb 4;11:3180. doi: 10.1038/s41598-021-82757-5 (PMC7862375; doi:10.1038/s41598-021-82757-5)
Supplement: Supplementary file 1 — Supplementary Information. [file 41598_2021_82757_MOESM1_ESM.docx]

**Near-infrared fundus autofluorescence alterations correlate with swept-source optical coherence tomography angiography findings in patients with Retinitis Pigmentosa.**

Marco Nassisi, Carlo Lavia, Saddek Mohand-Said, Vasily Smirnov, Aline Antonio, Christel Condroyer, Juliette Varin, Alain Gaudric, Christina Zeitz, José-Alain Sahel, Isabelle Audo.

**Supplementary material**

**Supplementary table. Available genotype for patients affected by retinitis pigmentosa enrolled in the study.**

| Patient ID | Gene (transcript) | Mutation | ACMG classification (criteria) | Reference |
| --- | --- | --- | --- | --- |
| CIC06851 | *USH2A* (NM_206933.4) | hetc.1036A>C, p.(Asn346His) | Uncertain significance (PM2,PP2,PP3,PP5) | Weston et al.^1^ |
|  | *USH2A* (NM_206933.4) | hetc.2276G>T, p.(Cys759Phe) | Uncertain significance (PM2,PP2,PP3,PP5) | Weston et al.^1^ |
| CIC03320 | *USH2A* (NM_206933.4) | hetc.4732C>T, p.(Arg1578Cys) | Uncertain significance (PM2,PP2,PP3,PP5) | Le Quesne Stabej et al.^2^ |
|  | *USH2A* (NM_206933.4) | hetc.10481C>G, p.(Thr3494Arg) | Uncertain significance (PM2,PP2,PP3) | This study |
| CIC08648 | *INPP5E* (NM_019892.6) | hetc.116del, p.(Pro39Hisfs*95) | Likely pathogenic  (PVS1,PM2) | This study |
|  | *INPP5E* (NM_019892.6) | hetc.746C>T, p.(Ser249Phe) | Uncertain significance (PM2,PP2,PP3) | This study |
|  | *INPP5E* (NM_019892.6) | hetc.1787G>C, p.(Arg596Thr) | Uncertain significance (PM2,PP2,PP3) | This study |
| CIC06732^ǂ^ | *USH2A* (NM_206933.4) | hetc.8609C>T, p.(Pro2870Leu) | Uncertain significance (PM2,PP2,PP3) | This study |
| CIC03709 | *USH2A* (NM_206933.4) | hetc. 2276G>T, p.(Cys259Phe) | Likely pathogenic  (PM2,PP1,PP2,PP3,PP5) | Weston et al.^1^ |
|  | *USH2A* (NM_206933.4) | hetc.15020C>T, p.(Pro5007Leu) | Uncertain significance (PM2,PP1,PP2,PP3) | Tiwari et al.^3^ |
| CIC07330^ǂ^ | *RPE65*  (NM_000329.3) | hetc.614A>G, p.(Asn205Ser) | Uncertain significance (PM2,PP2,PP3) | Morimura et al.^4^ |
| CICRCD1* | *MYO7A* (NM_001127180.2) | hetc.73G>A, p.(Gly25Arg) | Likely pathogenic (PS1,PM2,PP2,PP3,PP5) | Liu et al.^5^ |
|  | *MYO7A* (NM_001127180.2) | hetc.6028G>A, p.(Asp2010Asn) | Uncertain significance (PM2,PP2,PP3) | Zong et al.^6^ |
| CICRCD2* | *MYO7A* (NM_001127180.2) | hetc.73G>A, p.(Gly25Arg) | Likely pathogenic (PS1,PM2,PP2,PP3,PP5) | Liu et al.^5^ |
|  | *MYO7A* (NM_001127180.2) | hetc.6028G>A, p.(Asp2010Asn) | Uncertain significance (PM2,PP2,PP3) | Zong et al.^6^ |
| CIC08466 | *RHO*  (NM_000539.3) | hetc.316G>A, p.Gly106Arg | Pathogenic (PS1,PM2,PM5,PP2,PP3,PP5) | Fishman et al.^7^ |
| CIC09530 | *USH2A* (NM_206933.4) | hetc.2276G>T, p.(Cys759Phe) | Likely pathogenic (PM2,PM3,PP2,PP3,PP5) | Weston et al.^1^ |
|  | *USH2A* (NM_206933.4) | hetc.1876C>T, p.(Arg626*) | Pathogenic (PVS1,PM2,PP3,PP5) | Weston et al.^1^ |
| CIC06483 | *PRPH2* (NM_000322.5) | hetc.594C>G, p.(Ser198Arg) | Uncertain significance (PM2,PP2,PP3,PP5) | Ramsden et al.^8^ |
| CIC03619 | *AIPL1*  (NM_ 001033055.3) | hoc.184G>C p.(Gly62Arg) | Uncertain significance (PM2,PP3) | This study |
| 1394302 | *USH2A* | Not available^#^ | Not applicable | Not applicable |
| CIC09779 | *RPGR-ORF15* (NM_001034853.2) | hemic.2218G>T, p.(Glu740*) | Likely pathogenic  (PVS1,PM2) | Pelletier et al.^9^ |
| CIC10621 | *RPGR-ORF15* (NM_001034853.2) | hemic.2944del, p.(Glu982Lysfs*107) | Likely pathogenic  (PVS1,PM2) | Pelletier et al.^9^ |
| CIC11837 | *NR2E3* (NM_016346.4) | hetc.166G>A, p.(Gly56Arg) | Likely pathogenic (PS1,PM2,PP2,PP3,PP5) | Coppieters et al.^10^ |
| CIC04704 | *RHO*  (NM_000539.3) | hetc.1039C>G, p.(Pro347Ala) | Likely pathogenic (PM2,PM5,PP2,PP3,PP5) | Macke et al.^11^ |
| CIC06552^ǂ^ | *USH2A* (NM_206933.4) | hetc.2299del, p.(Glu767Serfs*21) | Pathogenic  (PVS1,PS3) | Lenassi et al.^12^ |
| CIC08647 | *EYS* (NM_001142800.2) | hetc.8267A>G, p.(Tyr2756Cys) | Uncertain significance (PM2,PM3,PP3) | This study |
|  | *EYS* (NM_001142800.2) | hetc.8628_8631del, p.(Asn2876Lysfs*5) | Pathogenic  (PVS1,PM2,PP3) | This study |
| CIC01532 | *USH2A* (NM_206933.4) | hetc.1256G>T, p.(Cys419Phe) | Uncertain significance (PM2,PP2,PP3,PP5) | Weston et al.^1^ |
|  | *USH2A* (NM_206933.4) | hetc.2299del, p.(Glu767Serfs*21) | Pathogenic  (PVS1,PS3) | Lenassi et al.^12^ |
| CIC10105 | *MAK* (NM_001242385.1) | hetc.1163G>C, p.(Ser388Thr) | Likely pathogenic (PM2,PM3,PP2,PP3) | This study |
|  | *MAK* (NM_001242385.1) | hetc.832-1G>C | Pathogenic  (PVS1,PM2,PP3) | This study |
| CIC10727 | *USH2A* (NM_206933.4) | hetc.2299del, p.(Glu767Serfs*21) | Pathogenic  (PVS1,PS3) | Lenassi et al.^12^ |
|  | *USH2A* (NM_206933.4) | hetc.485+1G>A | Pathogenic  (PVS1,PM2,PP3) | This study |

* Subjects are siblings. ^#^ Genetic results obtained in another center. ^ǂ^ Second allele is missing; ACMG: American College of Medical Genetics and Genomics. PVS1: Null variant (nonsense, frameshift, canonical ±1 or 2 splice sites, initiation codon, single or multiexon deletion) in a gene where loss of function is a known mechanism of disease. PS1: Same amino acid change as a previously established pathogenic variant regardless of nucleotide change. PS3: Well-established *in vitro* or *in vivo* functional studies supportive of a damaging effect on the gene or gene product. PM2: Absent from controls (or at extremely low frequency if recessive [≤0.5%]) in the Genome Aggregation Database (https://gnomad.broadinstitute.org). PM3: For recessive disorders, detected *in trans* with a pathogenic variant. PM5: Novel missense change at an amino acid residue where a different missense change determined to be pathogenic has been seen before. PP1: Cosegregation with disease in multiple affected family members in a gene definitively known to cause the disease. PP2: Missense variant in a gene that has a low rate of benign missense variation and in which missense variants are a common mechanism of disease. PP3: Multiple lines of computational evidence support a deleterious effect on the gene or gene product (≥ 2 different algorithms). PP5: Reputable source recently reports variant as pathogenic (≥ 3 publications from different groups and/or ClinVar classification as Pathogenic or Likely pathogenic [https://www.ncbi.nlm.nih.gov/clinvar/]).

**References**

1. Weston MD, Eudy JD, Fujita S, et al. Genomic structure and identification of novel mutations in usherin, the gene responsible for Usher syndrome type IIa. *Am J Hum Genet*. 2000;66(4):1199-1210. doi:10.1086/302855

2. Le Quesne Stabej P, Saihan Z, Rangesh N, et al. Comprehensive sequence analysis of nine Usher syndrome genes in the UK National Collaborative Usher Study. *J Med Genet*. 2012;49(1):27-36. doi:10.1136/jmedgenet-2011-100468

3. Tiwari A, Bahr A, Bähr L, et al. Next generation sequencing based identification of disease-associated mutations in Swiss patients with retinal dystrophies. *Sci Rep*. 2016;6:28755. doi:10.1038/srep28755

4. Morimura H, Fishman GA, Grover SA, Fulton AB, Berson EL, Dryja TP. Mutations in the RPE65 gene in patients with autosomal recessive retinitis pigmentosa or leber congenital amaurosis. *Proc Natl Acad Sci USA*. 1998;95(6):3088-3093. doi:10.1073/pnas.95.6.3088

5. Liu XZ, Newton VE, Steel KP, Brown SD. Identification of a new mutation of the myosin VII head region in Usher syndrome type 1. *Hum Mutat*. 1997;10(2):168-170. doi:10.1002/(SICI)1098-1004(1997)10:2<168::AID-HUMU10>3.0.CO;2-Y

6. Zong L, Chen K, Wu X, Liu M, Jiang H. Compound heterozygous MYO7A mutations segregating Usher syndrome type 2 in a Han family. *Int J Pediatr Otorhinolaryngol*. 2016;90:150-155. doi:10.1016/j.ijporl.2016.09.010

7. Fishman GA, Stone EM, Gilbert LD, Sheffield VC. Ocular findings associated with a rhodopsin gene codon 106 mutation. Glycine-to-arginine change in autosomal dominant retinitis pigmentosa. *Arch Ophthalmol*. 1992;110(5):646-653. doi:10.1001/archopht.1992.01080170068026

8. Ramsden SC, Davidson AE, Leroy BP, et al. Clinical utility gene card for: BEST1-related dystrophies (Bestrophinopathies). *Eur J Hum Genet*. 2012;20(5). doi:10.1038/ejhg.2011.251

9. Pelletier V, Jambou M, Delphin N, et al. Comprehensive survey of mutations in RP2 and RPGR in patients affected with distinct retinal dystrophies: genotype-phenotype correlations and impact on genetic counseling. *Hum Mutat*. 2007;28(1):81-91. doi:10.1002/humu.20417

10. Coppieters F, Leroy BP, Beysen D, et al. Recurrent mutation in the first zinc finger of the orphan nuclear receptor NR2E3 causes autosomal dominant retinitis pigmentosa. *Am J Hum Genet*. 2007;81(1):147-157. doi:10.1086/518426

11. Macke JP, Hennessey JC, Nathans J. Rhodopsin mutation proline347-to-alanine in a family with autosomal dominant retinitis pigmentosa indicates an important role for proline at position 347. *Hum Mol Genet*. 1995;4(4):775-776. doi:10.1093/hmg/4.4.775

12. Lenassi E, Saihan Z, Bitner-Glindzicz M, Webster AR. The effect of the common c.2299delG mutation in USH2A on RNA splicing. *Exp Eye Res*. 2014;122:9-12. doi:10.1016/j.exer.2014.02.018
